# Gadolinium-oxide nanoparticles for cryogenic magnetocaloric applications

**DOI:** 10.1038/s41598-022-06132-8

**Published:** 2022-02-10

**Authors:** A. Zeleňáková, P. Hrubovčák, A. Berkutova, O. Šofranko, N. Kučerka, O. Ivankov, A. Kuklin, V. Girman, V. Zeleňák

**Affiliations:** 1 Institute of Physics, P.J. Šafárik University, Park Angelinum 9, 04001 Košice, Slovakia; 2 Frank Laboratory of Neutron Physics, Joint Institute for Nuclear Research, Joliot-Curie 6, Dubna, Russia 141980; 3 Department of Physical Chemistry of Drugs, Comenius University in Bratislava, Mlynská dolina, 832 32 Bratislava, Slovakia; 4 Institute for Safety Problems of Nuclear Power Plants NAS of Ukraine, Kyiv, Ukraine; 5 Moscow Institute of Physics and Technology, Institutsky per. 9, Dolgoprudny, Moscow Region, Russia 141700; 6 Institute of Chemistry, Faculty of Science, P.J. Šafárik University, Moyzesova 11, 041 54 Košice, Slovakia

**Keywords:** Energy science and technology, Nanoscience and technology, Physics

## Abstract

The series of advanced nanocomposites consisting of Gd_2_O_3_ nanoparticles (NPs) embedded into periodic porous SiO_2_ matrix have been investigated with respect to their structural and magnetocaloric properties. By means of small angle neutron scattering and transmission electron microscopy, regular nanopores organized in the cubic or hexagonal superlattice have been documented. The pores are occupied by the NPs of progressive concentration within the nanocomposite series. All of the examined systems have exhibited extraordinarily high values of magnetic entropy change (up to 70 J kg^−1^ K^−1^) at low temperatures with the absence of thermal hysteresis, indicating their perspective utilization in cryogenic refrigeration. Profound analysis of magnetic entropy change data via scaling laws has been applied to the nanocomposite materials for the very first time. With the aid of scaling analysis, conclusions on magnetic properties and phase transition type have been made, even for the conditions unavailable in the laboratory.

## Introduction

**Scheme 1:**
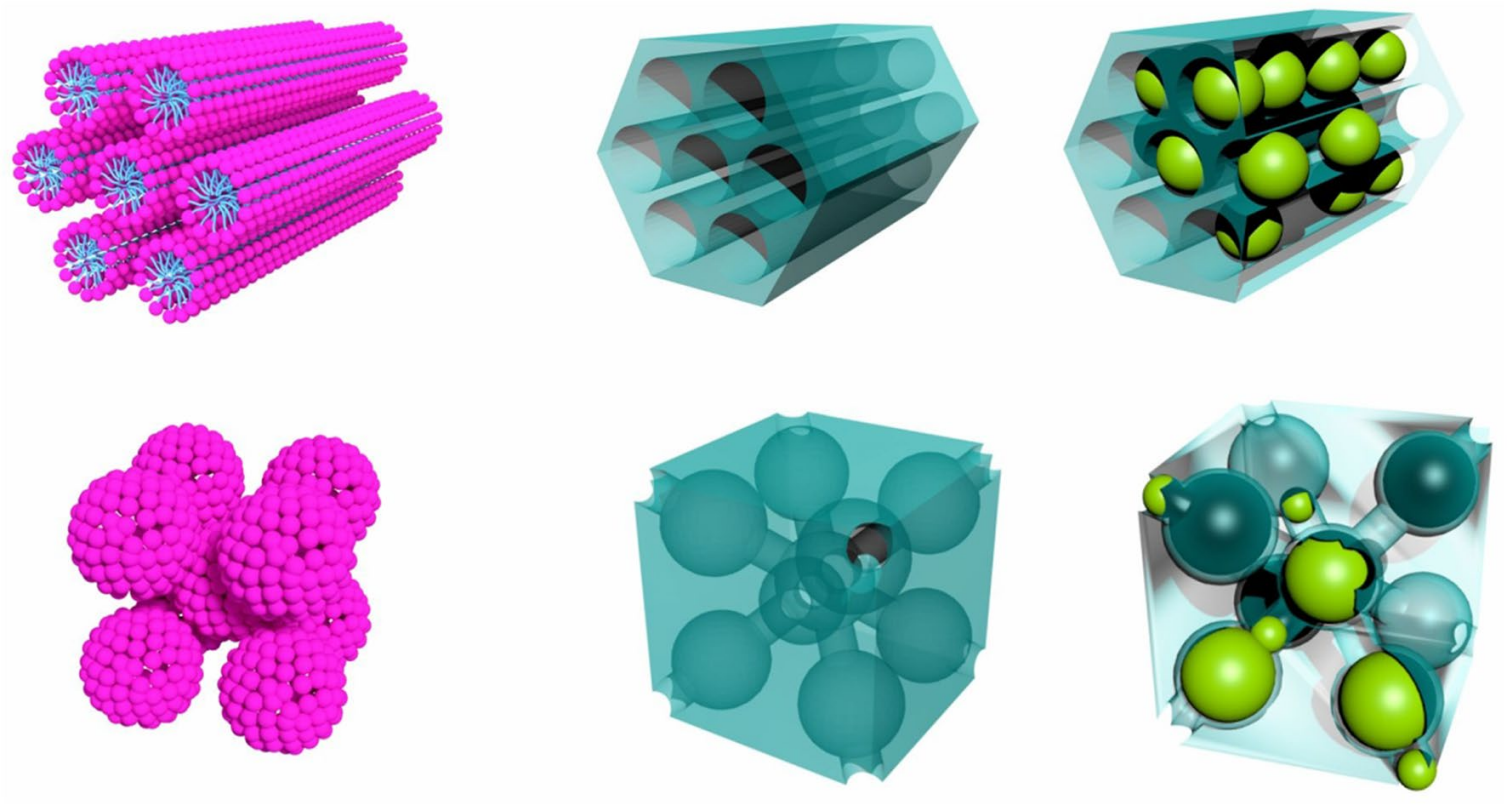
Illustration of the SiO_2_/Gd_2_O_3_ nanocomposite preparation and structure organization. Hollow silica matrices with regular pores organized into hexagonal (upper panel) and cubic (lower panel) superlattices were modified by the introduction of Gd_2_O_3_ nanoparticles (right-hand schemes).

The demand for eco-friendly technologies we are facing in recent years is the impetus for the development of advanced energy-efficient systems and devices. It is evident, in particular, in the scope of refrigeration, what is documented by an increasing number of related scientific publications^1–3^. It is expected that conventional technology based on gas expansion will be soon replaced by a fundamentally different principle exploiting magnetocaloric effect (MCE). MCE is related to magneto-thermodynamic phenomenon, i.e. a temperature change induced in a material by the variation of applied magnetic field. Even though the effect has been investigated over the century^4^, only the current progress in material science and nanotechnologies in particular facilitates its operative application up to room temperature^5,6^. Besides improved energy efficiency, low manufacturing costs, and negligible environmental impact, there is a number of scientific criteria that perspective magnetic refrigerant material has to meet. This is where nanoscale materials may manifest their unique benefits. The main advantage of nanoparticles over the bulk materials stems from wider options for tuning the characteristics of the refrigerant material. While the change of working temperature, refrigeration capacity or phase transition character in bulk materials can be induced almost exclusively by chemical composition, in the case of NPs, size, shape, capping layer or dilution of nanoparticle system affect those qualities. Moreover, it is assumed that nanomagnets could exhibit enhanced magnetic entropy change (*ΔS*_*M*_) because of high magnetic-moment density in a single magnetic particle. This is in fact observed in nanoparticles with large magnetic moments of individual atoms which may easily and coherently change their alignment even upon the application of a low magnetic field. Several compounds, particularly those based on rare earth (*RE*) elements, are therefore currently under investigation as promising candidates for significant MCE promotion. It is well known that *RE*^3+^ ions exhibit enhanced magnetic performance due to their specific electron configuration. If the electrons occupying partially unfilled 4f. shell are well localized, magnetic moments of *RE* could be quite large^7,8^. Usually, *RE* intermetallic compounds undergo magnetic transitions at low temperatures. However, their extensive scrutinization in recent years shows an ample variability in tuning their properties towards further applications. It has been found that TmZn manifests giant MCE even at lower applied field changes and its transition temperature and magnetic entropy change can be regulated by preassure^7^. The family of ternary *RE* ompounds manifest excellent MCE performance in wide temperature range depending on the specific composition and crystallic structure^7,8^. The composite systems exhibiting table-like MCE can be designed and prepared by their appropriate mixture. By this manner a composite covering wide working temperature range from 40 to 100 K has been proposed based on quaternary *RE* compounds containing La and Pr^9^. However, its drawbacks related to the thermal and magnetic hysteresis have yet to be overcome. Interestingly, even amorphous ribbons may exhibit large MCE, where − *ΔS*_*M*_ up to 18 J Kg^−1^ K^−1^ (at field change 5 T) has been reported for RE_60_Co_20_Ni_20_ (RE = Ho, Er) recently^10^. Among the most perspective *RE*-based candidates for MCE applications, gadolinium compounds are thoroughly investigated in particular^11–15^. We contribute to the scope with our study of gadolinium oxide nanocomposite series (see Scheme 1). The analysis of magnetic entropy change data by means of scaling laws allows for the determination of phase transition mechanism, which is in the case of nanoscale systems at low temperatures often very elusive. Although the employed scaling analysis has been established for bulk materials, we demonstrate its first valid application to the nanocomposite systems. The results presented in this work are set into the context of our profound experimental and theoretical analysis that we have carried out to the systems of this kind in recent years^16–22^. Altogether they provide a comprehensive insight into the nature and performance of advanced nanocomposites that we have prepared and examined.

## Results

### Structure and morphology

In order to examine the influence of nanoparticle abundance on nanocomposite properties, four SBA15/Gd_2_O_3_ systems with increasing concentration of Gd_2_O_3_ NPs (gadolinium precursor concentrations 0.01 M, 0.1 M, 0.5 M and 4 M) have been prepared and denoted as SBA15/Gd_2_O_3_-0.01 M, SBA15/Gd_2_O_3_-0.1 M, SBA15/Gd_2_O_3_-0.5 M and SBA15/Gd_2_O_3_-4 M. The structure of SBA15/Gd_2_O_3_ nanocomposite series has been investigated by means of small angle neutron scattering (SANS). Neutrons with their excellent penetration ability are recognized as an appropriate tool for the examination of structures hidden in the bulk material. Unlike the X-rays, neutrons are dominantly scattered by the nuclei and their interaction with electron shell of the atoms in the compound is negligible. The amplitude of neutron scattering varies independently of the element’s position in the periodic table and differs for isotopes of the same element. Due to this, neutron scattering offers a specific sensitivity to certain elements.

**Figure 1:**
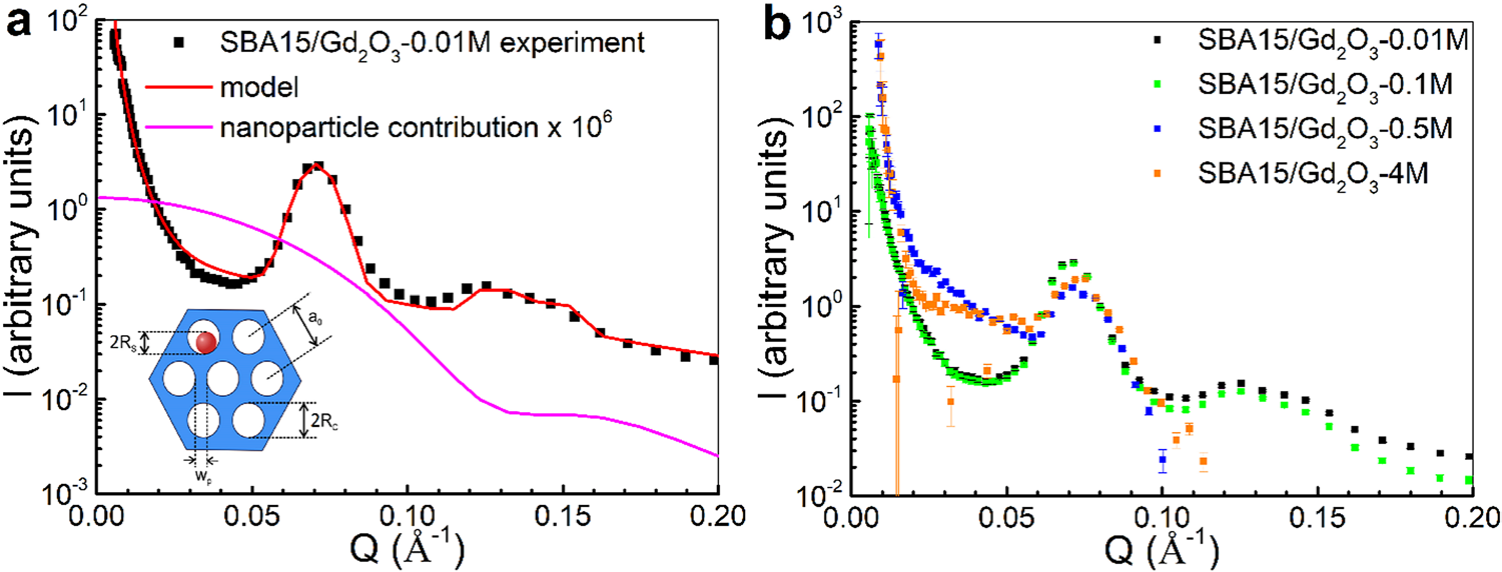
(**a**) The best model fit (red line) to the SANS experimental data (black squares) corresponding to the SBA15/Gd_2_O_3_-0.01 M composite. The magenta line represents a million-fold rescaled (facilitating the comparison) contribution of the polydisperse spheres (nanoparticles) to the total SANS intensity. Inset at the bottom left shows the schematics of nanocomposite with depicted structural parameters. (**b**) SANS data corresponding to the series of nanocomposites with increasing Gd_2_O_3_ nanoparticle concentrations (0.01, 0.1, 0.5 and 4 M) in the pores of SBA15 matrix.

Figure 1a shows SANS data obtained for the SBA15/Gd_2_O_3_-0.01 M composite. The system is characteristic of very low nanoparticle concentration what has been evidenced by a number of experimental methods (HRTEM, HE-XRD, nitrogen adsorption/desorption volumetry, magnetic measurements) previously^16,18^. The matrix pores are therefore deemed sparsely occupied by the Gd_2_O_3_ NPs and the scattering signal from the composite is supposed to closely resemble that of the hollow SBA15 matrix. In order to address the structure of the system, we have applied the model that we have developed for similar series of nanocomposites, where Fe_2_O_3_ instead of Gd_2_O_3_ NPs were introduced into the SBA15 matrix^17^. Briefly, total SANS intensity of the composite is assumed as a superposition of signals corresponding to the two subsystem contributions – scattering from the blank matrix and the system of Gd_2_O_3_ NPs. Employing the model, we have analyzed the SANS data of SBA15/Gd_2_O_3_-0.01 M (Fig. 1a) composite with the following results. As expected, the contribution of nanoparticle subsystem to the total scattering has been found about six orders of magnitude lower than the signal corresponding to the model of hollow silica matrix. Hence, features characteristic of the amorphous SBA15 matrix with regular cylindrical pores arranged in hexagonal symmetry (the schematics in the inset of the Fig. 1a) can be unequivocally recognized. It is the major peak at momentum transfer value *Q* ~ 0.07 Å^−1^, followed by the two less clearly resolved minor peaks at *Q* ~ 0.13 Å^−1^ and *Q* ~ 0.15 Å^−1^. These peaks attribute to the system of cylinders (pores) with average radius *R*_*c*_ ~ 4 nm (σ_c_ = 0.5) that are arranged in the regular lattice with parameter *a*_*0*_ ~ 9.8 nm (σ_a_ = 0.0006).

Figure 1b presents SANS data of our complete series of the examined SBA15 nanocomposites. One can notice the similarities between the *I(Q)* patterns corresponding to the two systems with a low concentration of gadolinium oxide NPs (i.e., 0.01 M and 0.1 M). Apparently, only a modest difference in the SANS data between the 0.01 M and 0.1 M systems suggests a low degree of Gd_2_O_3_ nanoparticles incorporation into the pores. To proceed further, let’s consider the changes in the *I(Q)* pattern of 0.5 M and 4 M systems with respect to those of low particle concentration. The first significant feature is the increase in the scattered neutron intensity in the range of 0.01 Å^−1^ < *Q* < 0.06 Å^−1^. This can be explained by the progressive occupation of the pores by the NPs. The elevated population of the Gd_2_O_3_ particles is reflected in the increase of its scattering contribution (see the magenta curve in Fig. 1a) to the total SANS signal. Since the pores are partially filled with NPs, the matrix pattern is becoming less resolved and the major peak at *Q* ~ 0.07 Å^−1^ smeared.

**Figure 2:**
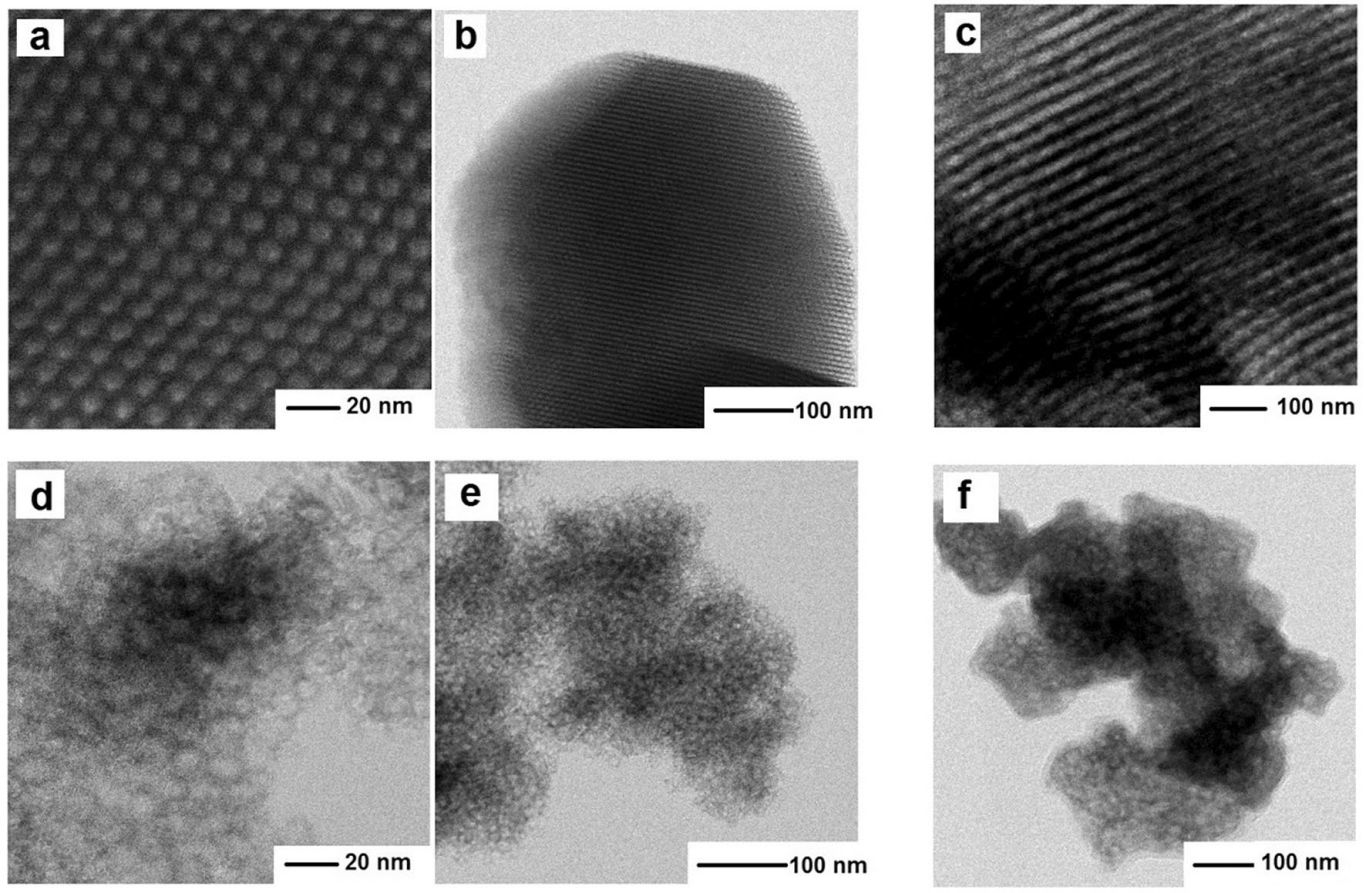
HRTEM micrographs depicting the SiO_2_/Gd_2_O_3_ nanocomposite structure organization for the systems with hexagonal (**a**–**c**) (upper panel) and cubic (**d**–**f**) (lower panel) symmetry. (**a**, **b**) represent blanck hexagonal matrix and **c** represents Gd_2_O_3_ nanoparticles in SBA15 matrix. (**d**, **e**) represent blank cubic matrix and (**f**) Gd_2_O_3_ nanoparticles in SBA16 matrix.

The presence of Gd_2_O_3_ nanoparticles within the matrix pores is evidenced by the transmission electron microscope (TEM) images in Fig. 2. The periodic structure with hexagonal symmetry (upper panel) and cubic symmetry (lower panel) is documented by the high resolution transmission electron microscopy (HRTEM) in STEM mode. The unit cell parameter of the hexagonal structure in SBA15/Gd_2_O_3_-4 M matrix has been estimated from the HRTEM micrographs to *a*_*0*_ ~ 12 nm, while *a*_*0*_ ~ 15 has been attributed to the cubic system SBA16/Gd_2_O_3_-4 M. Hexagonal and cubic pore diameters have been determined to be *d*_*SBA15*_ ~ 7 nm and *d*_*SBA16*_ ~ 10 nm, respectively, in good agreement with references^22,23^.

**Figure 3:**
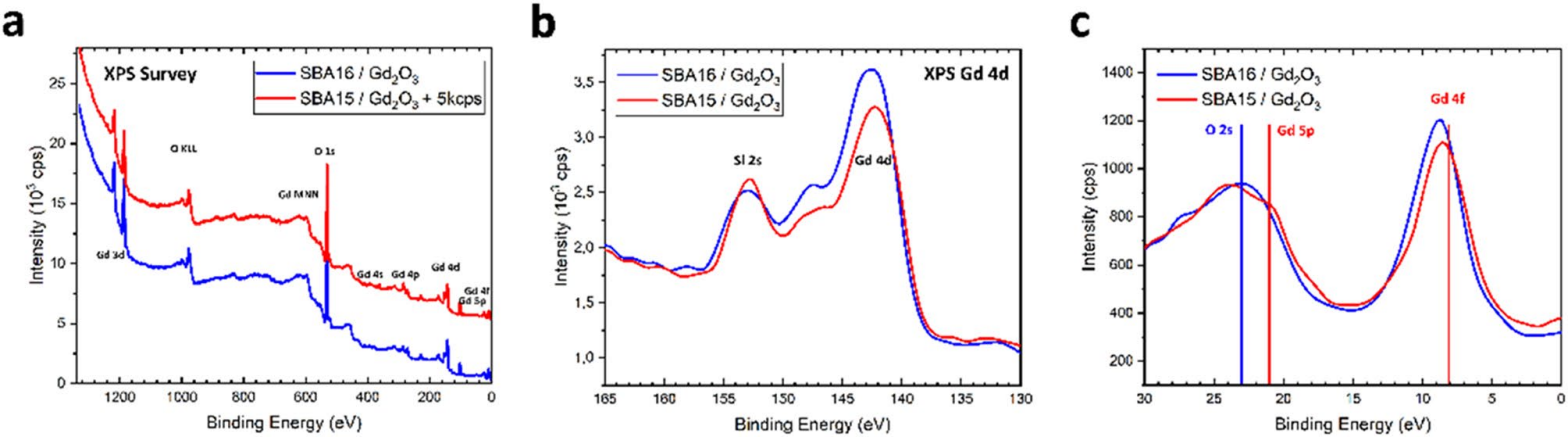
(**a**) Survey XPS spectrum of the SBA16/Gd_2_O_3_ (cubic) and SBA15/Gd_2_O_3_ (hexagonal) nanocomposites, (**b**) XPS core-level spectra from Gd_2_O_3_ (cubic) and Gd_2_O_3_ (hexagonal), (**c**) XPS valence bands (VB) mapping of Gd_2_O_3_ (cubic) and Gd_2_O_3_ (hexagonal).

The survey X-ray photoelectron spectroscopy (XPS) spectrum is shown in Fig. 3a. The results of the XPS survey confirm the chemical composition of studied SBA15/Gd_2_O_3_ and SBA16/Gd_2_O_3_ samples without additional chemical contamination. This verifies the existence of Si, Gd, and O atoms only in studied samples. The core-level spectra from XPS, Fig. 3b, clearly exhibit essentially broader Gd 4d peaks in core-level spectra. The valence band (VB) area up to 30 eV was recorded for the samples and compared with that of the Gd-Ox XPS external standard (see Fig. 3c). The range of binding energies (BE) from 18 to 24 eV usually belongs to the O 2 s electronic core-like states for the case of simple oxides, vertical blue line. The band at 22.51 eV is from the partial contribution from Gd 5*p* due to band overlap. The manifestation of the strongly asymmetric shoulder at 8.5 eV (see Fig. 3c, red line) allows us to assume that this is also from the Gd 4*f* electronic states. XPS analysis confirms the presence of Gd_2_O_3_ nanoparticles without any further contamination.

### Magnetic entropy study and scaling analysis

**Figure 4:**
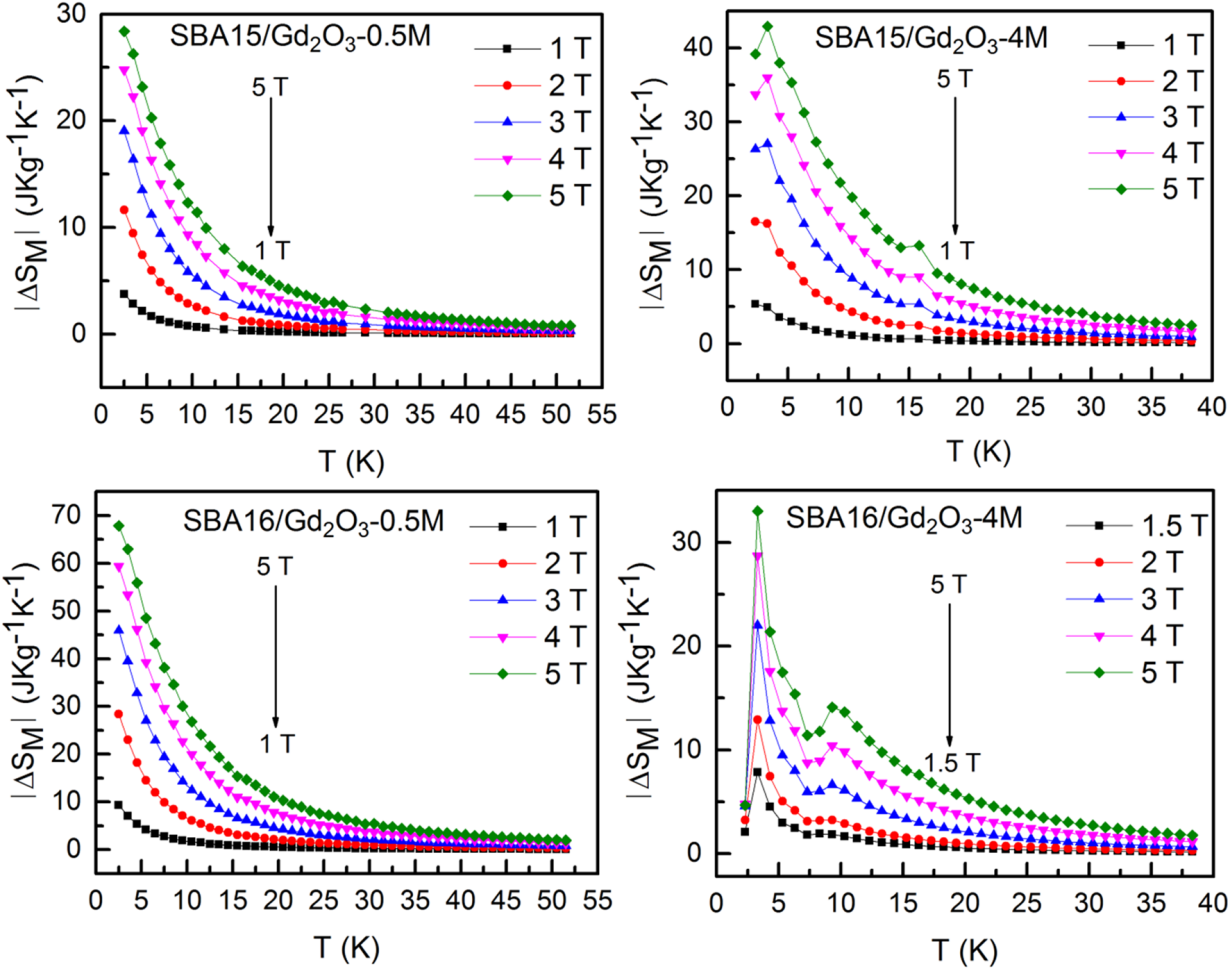
Magnetic entropy change of the examined nanocomposites calculated from experimental *M(H,T)* data for the applied field change up to 5 T.

The magnetocaloric effect has been evaluated in the series of nanocomposites with higher nanoparticle concentration, i.e. 0.5 M and 4 M, in both SBA15 and SBA16 matrix types. Figure 4 shows the temperature dependence of magnetic entropy change − *ΔS*_*M*_ calculated for the examined systems according to Eq. (1) (Methods section) for the field change from 0 to 5 T. All of the systems exhibit typical paramagnetic behavior with abrupt − *ΔS*_*M*_*(T)* increase when approaching low temperatures. The only exception is SBA16/Gd_2_O_3_-4 M composite, where two peaks of − *ΔS*_*M*_*(T)* dependences at *T* ~ 3 K and *T* ~ 9 K have been recognized.

**Figure 5:**
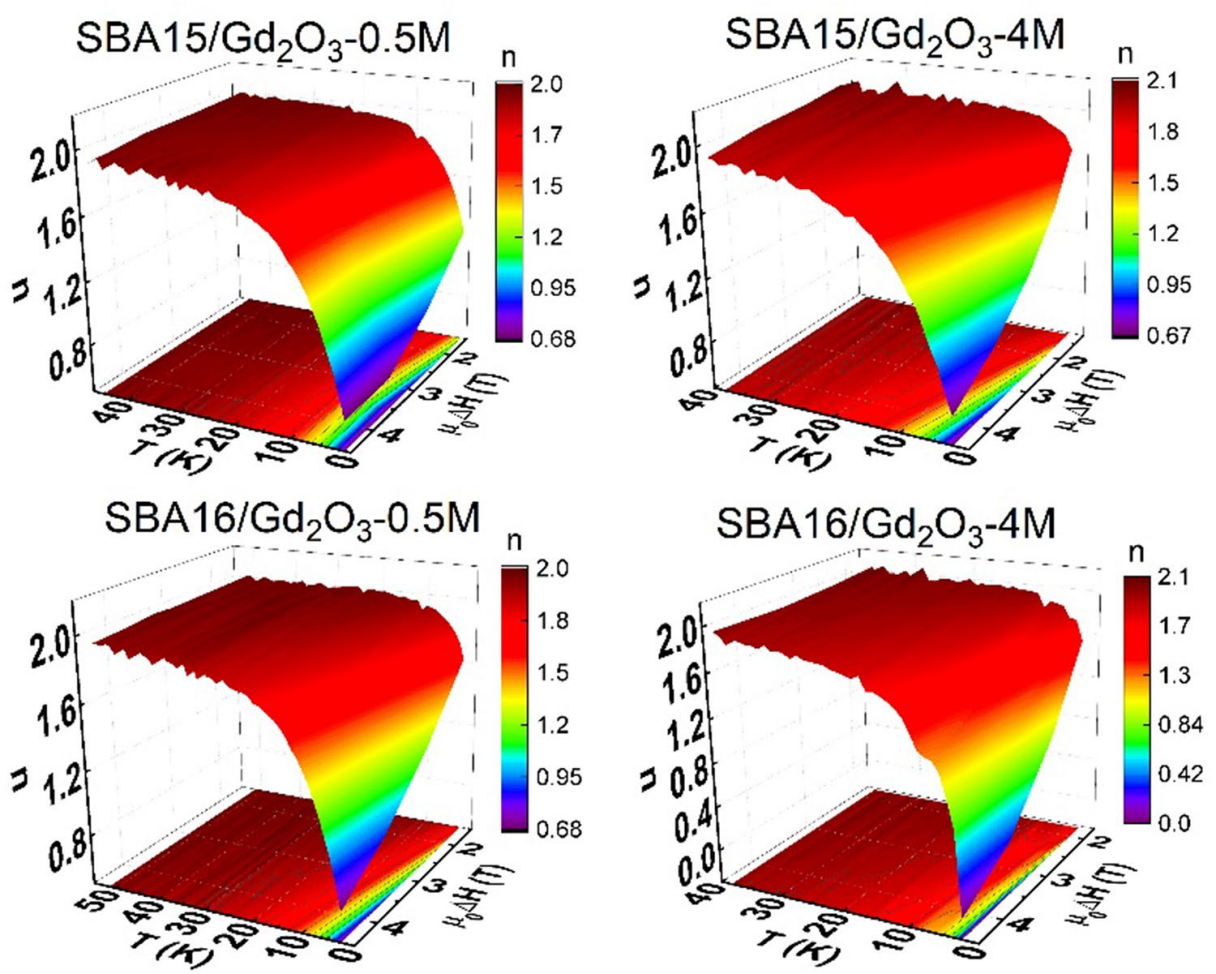
Temperature dependence of *n* index in the studied nanocomposite systems.

Apparently, there is a lack of data at low temperature region for the clear − *ΔS*_*M*_*(T)* peak recognition. Consequently, the direct application of scaling analysis was not possible and the universal curve had to be constructed alternatively. For this purpose, the exponent *n* characterizing applied field dependence of *ΔS*_*M*_ ∝ *H*^*n*^ has been calculated according to the Eq. (2) and the data are displayed in Fig. 5.

**Figure 6:**
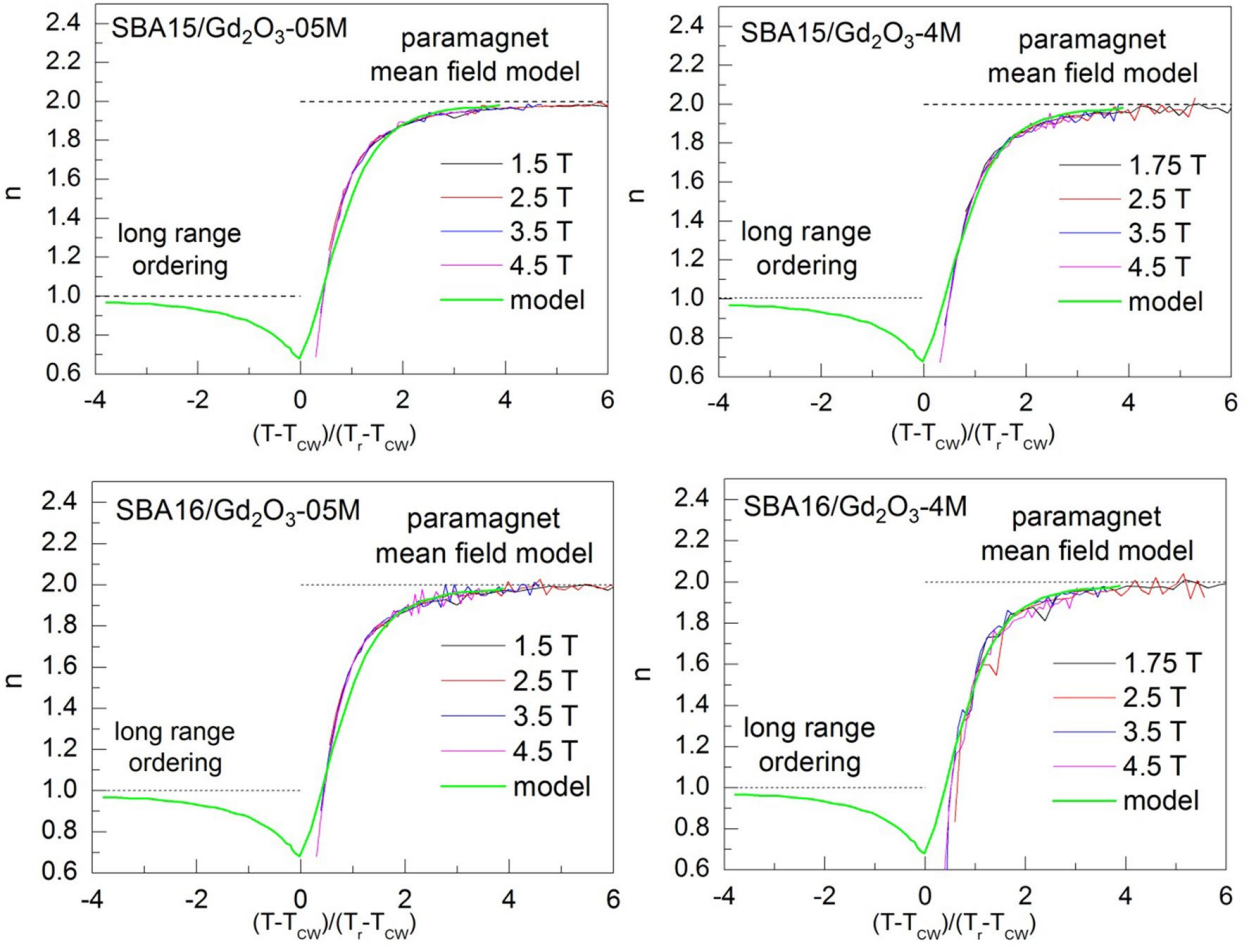
Universal curves constructed for *n(T)* data and their comparison to the SOPT according to the mean field model.

The common feature of all the examined systems is a steep increase of *n* at low temperatures (up to *T* ~ 9 K) while in the higher temperature region it converges to the value *n* = 2 regardless of applied field magnitude. One can also recognize that data are noisy when approaching higher temperatures (*T* > 25 K). This is a consequence of low field dependence of *ΔS*_*M*_ in the region (significant thermal fluctuations) that is the source of inaccuracy when calculating logarithm and differentiating according to the Eq. (2). In the case of the second order phase transition (SOPT), *n(T,H)* curves should collapse onto universal curve after rescaling temperature axis (Section Methods). Due to the absence of a peak in the both −*ΔS*_*M*_*(T)* (except of SBA16/Gd_2_O_3_-4M) and its corresponding minimum in *n(T)* data (all the systems), we have determined the *T*_*CW*_ = − 2 K from the Curie–Weiss law fit to the magnetic susceptibility data (Supplementary Figure 1). Further, *T*_*r*_ has been selected for each branch of *n(T)* as temperature corresponding to *n* = 1.62 for the composites SBA15/Gd_2_O_3_-0.5 M, SBA16/Gd_2_O_3_-0.5 M and *n* = 1.55 for SBA15/Gd_2_O_3_-4 M and SBA16/Gd_2_O_3_-4 M (Supplementary Figure 2). The values have been set arbitrarily, although with the requirement of the proximity to the expected phase transition. The collapse of the data along with its comparison to the SOPT model of mean field theory^24^ can be seen in the Fig. 6.

**Figure 7:**
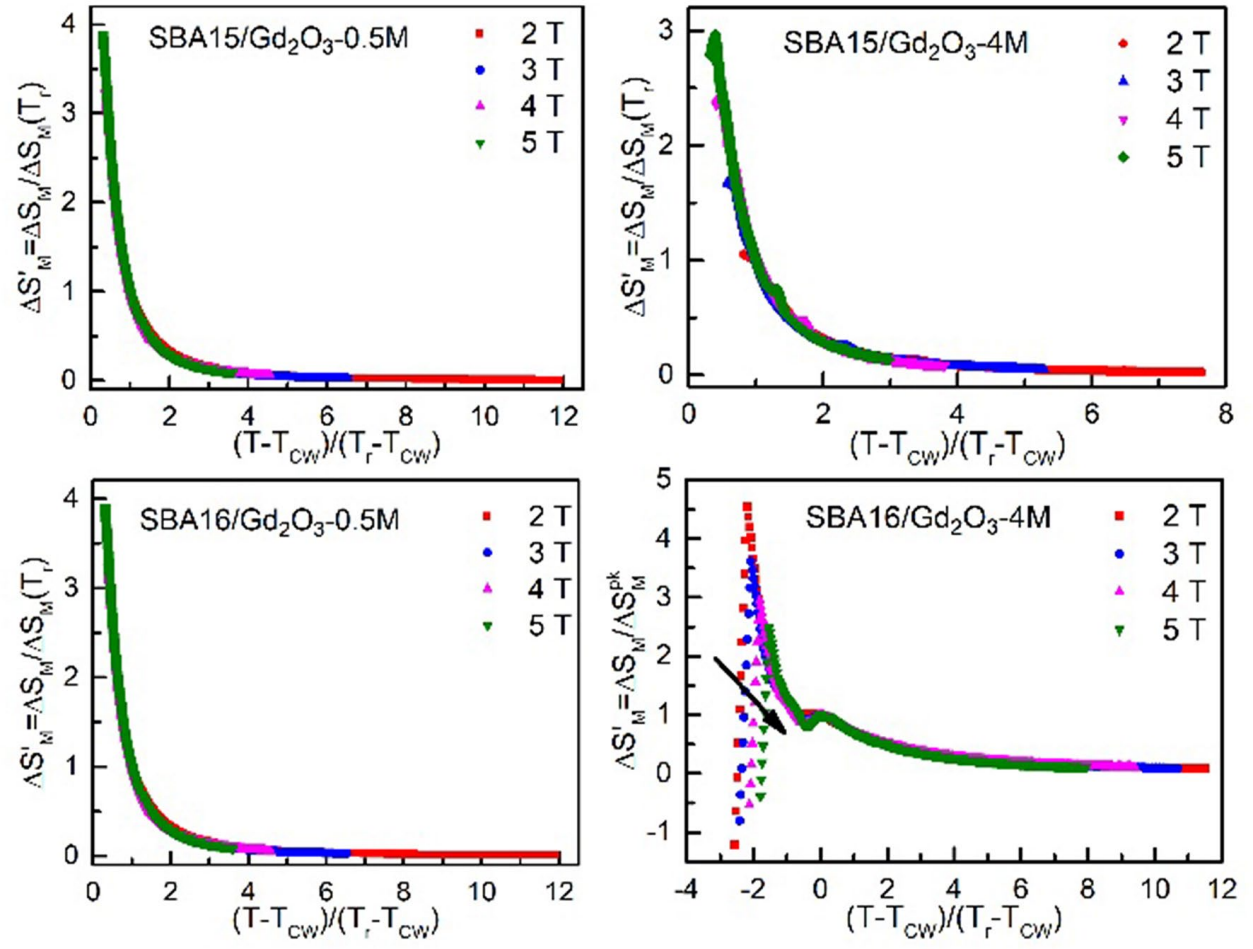
Collapse of magnetic entropy change onto the universal curve utilizing parameters derived from *n(T,H)* analysis of SBA15/Gd_2_O_3_-0.5 M, SBA16/Gd_2_O_3_-0.5 M and SBA15/Gd_2_O_3_-4 M. Scaling of the SBA16/Gd_2_O_3_-4 M has been performed directly employing the parameters obtained from − *ΔS*_*M*_*(T)* peak at *T* ~ 9 K.

Employing rescaled temperature axis, the collapse of *ΔS*′_*M*_ = *ΔS*_*M*_*/ΔS*_*M*_*(T*_*r*_*)* has been obtained for the systems SBA15/Gd_2_O_3_-0.5 M, SBA16/Gd_2_O_3_-0.5 M and SBA15/Gd_2_O_3_-4 M, Fig. 7 (section Discussion). Due to the presence of minor peak (*T* ~ 9 K) in the − *ΔS*_*M*_* (T)* data, the collapse has not been achieved in the case of SBA15/Gd_2_O_3_-4 M. Therefore, an alternative (standard) method has been applied^24^. We assigned *T*_*CW*_ to the peak value − *ΔS*^*pk*^_*M*_ at *T* ~ 9 K. Magnetic entropy change values have been rescaled according to *ΔS*′_*M*_ = *ΔS*_*M*_*/ΔS*^*pk*^_*M*_ while temperature axis with respect to *T*_*r*_ that corresponds to the *ΔS*_*M*_*(T*_*r*_*)* = *0.7ΔS*^*pk*^_*M*_. The rescaled data are shown in Fig. 7. Apparently, the universal curve has been obtained on the right side of the minor peak, whereas in the lower temperature region the scaling failed.

## Discussion

Detailed analysis of the Gd_2_O_3_ particles’ abundance in the matrix by means of SANS is hampered by the fundamental drawback that is inherent to the gadolinium nuclei. Gd is recognized as the element with by far the highest thermal neutron capture cross section among all stable isotopes^25^. Evidence on enhanced neutron absorption of the systems with higher Gd_2_O_3_ nanoparticle abundance (4 M vs. 0.5 M) is documented clearly in the *Q*-range over 0.1 Å^−1^, Fig. 1. Despite of pronounced absorption effect, the fingerprints corresponding to the structural features of SBA15 matrix have been resolved (peaks at *Q* ~ 0.07, 0.13 and 0.15 Å^−1^), while its gradual smearing with increasing nanoparticle concentration suggests the progressive occupation of the pores by Gd_2_O_3_ NPs. As it has been evidenced in the case of nanocomposite series containing Fe_2_O_3_ NPs^22^, matrix symmetry significantly affects magnetic performance of the system. For the equivalent nanoparticle concentration, stronger inter-particle interactions (hallmarks of superspin glass) have been documented in the systems with cubic matrix, while weak dipolar interactions have been found in the hexagonal matrix. This behavior has been ascribed to the larger distances among the NPs in the latter series. In the case of Gd_2_O_3_ NPs, the effect is not observed. In fact, SiO_2_/Gd_2_O_3_ nanocomposites do not exhibit superparamagnetic, but pure paramagnetic properties. It is evidenced by the ZFC–FC magnetization data^16^, Supplementary Figure S1, where the absence of thermal hysteresis (irreversibility of the curves) along with the absence of ZFC peak have been manifested. Hence, we can conclude that magnetic properties of SiO_2_/Gd_2_O_3_ are determined by the magnetic moments of single atoms and not by the collective behavior of nanoparticle superspins like in nanocomposite series SiO_2_/Fe_2_O_3_^22^. The presence of inter-particle interactions may suppress magnetocaloric performance of the system. Since mutualy interacting moments are less susceptible to follow applied magnetic field change, the magnitude of magnetic entropy change may decrease accordingly. The absence of inter-particle interactions and thermal hysteresis therefor can be assumed as significant factors leading to enhanced MCE observed in our SiO_2_/Gd_2_O_3_ nanocomposites and suggests them for possible magnetic refrigeration applications.

The support of successful filling process of porous matrix system with NPs is provided also by the magnetization vs temperature data^16^. With the increasing concentration of Gd_2_O_3_ NPs in nanocomposites with hexagonal symmetry, the magnetization measured at corresponding temperatures is gradually enhanced. On the other hand, the magnetization curves recorded in nanocomposites with cubic symmetry show that presumably there is a critical precursor concentration limit for the nanoparticle nucleation within the pores. This would explain unexpected higher magnetization values bellow this critical limit and lower magnetization values beyond the limit. This suggests a scenario that in the case of cubic nanocomposites, certain fraction of Gd_2_O_3_ is evicted out from internal surface pores during the matrix modification by the precursor of high concentration (apparently above 0.5 M).

The analysis of magnetic susceptibility vs temperature data revealed typical paramagnetic behavior of all the examined systems at higher temperatures (Supplementary Figure 1). Although steep increase of − *ΔS*_*M*_ is expected for the paramagnetic salts when approaching low temperatures, the values obtained at ~ 2 K for the field change 5 T in our nanocomposites (above ~ 30 J Kg^−1^ K^−1^) are remarkably high, Fig. 4. Among the family of gadolinium oxides, the candidates that would approach that level of − *ΔS*_*M*_ under comparable conditions (T ~ 2 K, *µ*_*0*_*ΔH* = 5 T ) are extremaly scarce. Recently, a series of works devoted to *RE* bulk compounds with orthorhombic double perovskite type structure have been published. Regarding magnetocaloric properties, the most perspective of them appear those containing gadolinium atoms Gd_2_ZnMnO_6_^26^, Gd_2_FeAlO_6_^27^ and Sr_2_GdNbO_6_^28^ with the largest − *ΔS*_*M*_ ~ 26 J Kg^−1^ K^−1^ determined for the latter compound. From nanoscaled structures, it is worth to mention Eu doped Gd_2_O_3_ nanorods^29^ that exhibit enhancened MCE when compared with the pure Gd_2_O_3_ nanotubes^30^. Here, the promotion of magnetic entropy change up to 20 J Kg^−1^ K^−1^ has been ascribed to the surface spin disorder. Apparently, at present none of these systems exceeds the qualities of our nanocomposites. Neither in magnetic entropy change magnitudes, nor in the potential working temperature range. In particular, magnitude ~ 70 J Kg^−1^ K^−1^ observed in the composite SBA16/Gd_2_O_3_-0.5 M belongs to the group of the highest |*ΔS*_*M*_| ever reported^31^. Undoubtedly, the present phenomenon deserves profound scrutiny in order to determine its nature. For this purpose we employed scaling analysis proposed by Franco et al.^24^ (Section Methods) that is based on the collapse of the − *ΔS*_*M*_* (T,ΔH)* data onto single universal curve. The application of magnetic entropy scaling laws may deliver information on the type of the phase transition, the presence of interactions or multiple phases in examined system. However, direct utilization of the method is hampered by the absence of a peak in the − *ΔS*_*M*_*(T,ΔH)* data corresponding to our nanocomposites (except of SBA16/Gd_2_O_3_-4 M). An alternative method for the universal curve construction therefor has been employed. The exponent *n(T,H)* derived from magnetic entropy change vs temperature data also collapse onto single master curve. The universal curve characteristic of the SOPT calculated in terms of the mean field theory model is shown in the Fig. 6 (green line) along with rescaled *n(T,H)* data of our nanocomposites. Very good agreement of rescaled data with the SOPT model for the three systems SBA15/Gd_2_O_3_-0.5 M, SBA16/Gd_2_O_3_-0.5 M and SBA15/Gd_2_O_3_-4 M has been achieved, while in the case of SBA16/Gd_2_O_3_-4 M the collapse is not sufficiently plausible. Nevertheless, the exponent characterizing field dependence of magnetic entropy change *ΔS*_*M*_ ∝* H*^*n*^ approaches the value of *n* = 2 at higher temperatures, what is common for the all examined nanocomposites. In this temperature region, we assume paramagnetic behavior of the systems following Curie–Weiss law *M* = *CH/(T *− *T*_*CW*_*)*, where *C* is Curie–Weiss constant. Application of the Eq. (1) to the Curie–Weiss law yields *ΔS*_*M*_ ~ *H*^*2*^ what in fact we observe. Recent works show that *n(T,H)* dependence can be utilized as a reliable quantitative criterion for determining the order of magnetic phase transitions. It has been theoreticaly justified and experimentaly evidenced in the series of compound families^32^ that overshooting of *n* = 2 value is one of the hallmarks unequivocally corresponding to the first order magneto-structural phase transitiuon (FOPT). Employing the criterion, FOPT has been recognized in RE_6_Co_2_Ga compounds when RE = Ho, Dy, wherease only SOPT has been observed in the case of introducing Gd^33^. Accurate determination of the phase transition nature is important both from fundamental and application point of view. Thermal hysteresis accompanying FOPT affects the cyclicality of the compound performance. Further very valuable benefit of scaling analysis is its ability to extrapolate the data to conditions not available in the laboratory. In the case of the systems SBA15/Gd_2_O_3_-0.5 M, SBA16/Gd_2_O_3_-0.5 M and SBA15/Gd_2_O_3_-4 M, it allows us for concluding on magnetic phase transition character that apparently occurs at negative temperature *T*_*CW*_ ~ − 2 K. According to the mean field model of SOPT^24^, *n* has a minimum at *T* = *T*_*CW*_ and its value is *n* = *2/3.* The comparison of our collapsed data with the model in the vicinity of critical temperature shows a rather good agreement. Therefor one can conclude on correct determination of *T*_*CW*_ ~ − 2 K and the long range magnetic ordering below the *T*_*CW*_ for the three systems although experimental confirmation is by nature impossible. On the other hand, the collapse of SBA16/Gd_2_O_3_-4 M data is apparently not conclusive, Fig. 6. The scaling was unsuccessful due to the presence of two − *ΔS*_*M*_*(T*) peaks. Their superimposition hinders determination of the correct *T*_*r*_ value. For the *n(T)* data corresponding to lower or higher field change *T*_*r*_ is attributed to different *T* ~ 3 K or *T* ~ 9 K maximum (Supplementary Figure 2). Taking into account that the peak occurrence in − *ΔS*_*M*_*(T*) and corresponding minimum in *n(T)* data is a signature of relaxation process^24^, one should treat each of the maxima separately with respective scaling parameters *T*_*CW*_, *T*_*r*_ and − *ΔS*^*pk*^_*M*_. Based on the analogy to the rest of our nanocomposites from the series, we can ascribe low temperature maximum (*T* ~ 3 K) to the SOPT. Indeed, we concluded on the SOPT in our earlier work devoted to the SBA16/Gd_2_O_3_-4 M system^19^. Hence, elucidation of the nature of the second − *ΔS*_*M*_*(T)* peak (*T* ~ 9 K) that emerges only in the SBA16/Gd_2_O_3_-4 M system from the series is yet to be done. Characteristic feature of the second peak is its onset at *Δµ*_*0*_*H* ~ 1.5 T and progressive promotion with higher applied field change. Likewise behavior is observed also for the respective minimum in the *n(T,H)* data (Supplementary Figure 2). This is in contradiction with the mean field theory^24^ where *n(T)* minimum is field independent attaining constant value *n* = 2/3 at the critical temperature *T*_*CW*_. However, according to Franco et al.^24^, this peculiar behavior may be indicative of multiple magnetic phase presence in the system. Therefore, − *ΔS*_*M*_*(T,ΔH)* data of SBA16/Gd_2_O_3_-4 M have been rescaled with respect to the second peak using corresponding parameters obtained directly from − *ΔS*_*M*_, Fig. 7.

The universal curve has been constructed for the data on the right side of the second peak, whereas in the lower temperature region scaling failed. The data exhibit typical signature of the minor magnetic phase presence in the system described by Franco et al.^34^, Fig. 7, where the arrow indicates the evolution of the curves with increasing magnetic field. The evidence of multiple magnetic phase coexistence is manifested also by the wasp-waist hysteresis loop at 2 K observed only for SBA16/Gd_2_O_3_-4 M from the nanocomposite series^16^. Despite of a peak absence, master curves have been obtained even in the magnetic entropy change data of SBA15/Gd_2_O_3_-0.5 M, SBA16/Gd_2_O_3_-0.5 M and SBA15/Gd_2_O_3_-4 M, Fig. 7. It was facilitated by the rescaled temperature axis that we adopted from the *n(T)* scaling analysis, Fig. 6. No signatures of multiple magnetic phase presence in the three systems have been recognized. Although the evidence on the physical nature of the − *ΔS*_*M*_*(T)* peak at *T* ~ 9 K in the SBA16/Gd_2_O_3_-4 M has been gathered, an unambiguous determination of its origin is difficult. Based on the enhanced neutron absorption apparent from SANS data (Fig. 1b) along with analogy to similar nanocomposite^16,35^ containing iron oxide NPs instead of Gd_2_O_3_, larger nanoparticles’ formation on the surface of the SBA16/Gd_2_O_3_-4 M nanocomposite is presumable. One can hypothesize on the coexistence of magnetic phases corresponding to the larger nanoparticles with higher crystallinity and small particles in the matrix pores with significant lattice disorder. In fact, crystalline Gd_2_O_3_ phase has been experimentally evidenced by X-ray diffraction only in the case of SBA16/Gd_2_O_3_-4 M system from the series^16^. However, due to the complexity of the examined nanocomposite systems, any conclusion on the phase composition without extra supporting data would be speculative.

## Conclusions

Based on the analysis of experimental data provided by SANS, HRTEM, XPS and magnetometry along with the context of results obtained for the likewise systems earlier, the following conclusions regarding the examined nanoporous SiO_2_/Gd_2_O_3_ nanocomposite series have been made. During matrix modification, progressive occupancy of the pores by the nanoparticles with increasing gadolinium precursor concentration was confirmed. In the case of the matrix with cubic symmetry (SBA16), a scenario of critical precursor concentration above 0.5 M was evidenced. All of the systems exhibited extraordinarily large values of magnetic entropy change at low temperatures that were nanoparticle concentration and matrix type dependent. Application of scaling analysis that was modified and applied to the nanocomposite systems for the first time revealed the nature of the enhanced magnetocaloric effect observed in the investigated series. With the aid of scaling laws, signatures of the second order phase transition from paramagnetic to long range ordered (antiferromagnetic) phase were determined, although the conditions were unavailable in the laboratory. The combination of unique magnetocaloric performance (SOPT, high − *ΔS*_*M*_*(T)*, absence of thermal and magnetic hysteresis) along with nanoscale dimensions that facilitate a low cost production of the presented nanocomposites favor our SBA15/Gd_2_O_3_ and SBA16/Gd_2_O_3_ systems for cryogenic magnetocaloric applications.

## Methods

### SANS technique

Small angle neutron scattering (SANS) measurements were carried out at the IBR-2 pulsed reactor in Frank Laboratory of Neutron Physics, Joint Institute for Nuclear Research in Dubna (Russia). The wavelength distribution of the incident thermal neutron beam resembles a modified Maxwell distribution ranging from ~ 0.2 to 6 Å with a spectral distribution maximum at ~ 1.8 Å. A small-angle time-of-flight spectrometer YuMO was used, allowing to cover the scattering vector *Q* range 0.006–0.6 Å^−1^ (*Q* = (4π/λ)sinθ, where λ is the neutron wavelength and 2θ is the scattering angle)^36^. Samples in powder form were loaded in aluminum cells and exposed to neutrons for approximately one hour at room temperature. The scattering from an empty cell was measured and subtracted from the sample scattering. The averaged scattering patterns were corrected for detector efficiency absorption, solvent scattering, and instrumental background. Raw data treatment was performed by the SAS software^37^.

### XPS technique

The X-ray photoelectron spectroscopy (XPS) spectra were measured at the constant pass energy of 50 eV. The energy scale of the spectrometer was calibrated by setting the measured Au 4*f* 7/2 and Cu 2p 3/2 binding energies to 84.00 ± 0.05 eV and 932.66 ± 0.05 eV, respectively, in reference to Fermi energy *E*_*F*_. The energy drift due to charging effects was calibrated, taking the XPS C1*s* (284.6 eV) core level spectrum of hydrocarbons as it was suggested for dielectric materials.

### Magnetic measurements

Magnetic measurements were performed by SQUID based magnetometer MPMS 5XL (Quantum Design). Powder samples of the nanocomposite systems were encapsulated in a gelatine capsule and fixed in the plastic straw. The signal contribution of the empty gel cap and the straw was subtracted from the total signal and the data were corrected for the diamagnetic contribution using Pascal’s constants. Isothermal magnetization data were obtained as follows. The sample was cooled in the absence of applied magnetic field down to initial temperature. Then the magnetization vs increasing applied field *M(H)* was recorded up to 5 T. Further, the temperature was elevated, fixed to higher value (up to 50 K) and another branch of *M(H)* was taken. Magnetic susceptibility was calculated from magnetization vs temperature (1.8–300 K) data obtained in zero field cooling (ZFC) regime under applied static field 100 Oe during progressive sample heating.

### Magnetocaloric properties

Magnetocaloric properties of the systems have been evaluated from the series of magnetization data. The collected *M(H,T)* data have been processed assuming the Maxwell relation^38^ *μ*_*0*_*(∂M/∂T)*_*H*_ = *(∂S/∂H)*_*T*_ that yields the expression for magnetic entropy change *ΔS*_*M*_ at applied field change from *H*_*1*_ to *H*_*2*_

1 $$\Delta {S}_{M}={\mu }_{0}{\int }_{{H}_{1}}^{{H}_{2}}{\left(\frac{\partial M}{\partial T}\right)}_{H}dH,$$

where *μ*_*0*_ is the free-space magnetic permeability. In practice, the partial derivative was replaced by finite differences and the experimental data were integrated numerically^19^. *ΔS*_*M*_*(T,ΔH)* data have been employed for further analysis where the local values of the exponent *n* characterizing the field dependence of *ΔS*_*M*_ ∝ *H*^*n*^ has been calculated as^39^

2 $$n=\frac{\partial ln\left|\Delta {S}_{M}\right|}{\partial H}.$$

Again, the partial derivative was replaced by finite differences during data procession. The collapse of *n(T,H)* dependences onto universal curve has been performed by rescaling temperature axis to relative temperature^39^ *θ* = *(T *− *T*_*CW*_*)/(T*_*r*_ − *T*_*CW*_*)*, where *T*_*CW*_ is Curie–Weiss temperature and the reference temperature *T*_*r*_ corresponds to the certain value of *n(T)* selected arbitrarily^24^. Once the rescaled temperature axis is constructed, the normalization of the *ΔS*_*M*_ curves can be done using^24^ *ΔS*′_*M*_ = *ΔS*_*M*_*/ΔS*_*M*_*(T*_*r*_*)*.

## Supplementary Information

Supplementary Information.
