# Supplementary material for: Gadolinium-oxide nanoparticles for cryogenic magnetocaloric applications
Source: Sci Rep. 2022 Feb 10;12:2282. doi: 10.1038/s41598-022-06132-8 (PMC8831503; doi:10.1038/s41598-022-06132-8)
Supplement: Supplementary file 1 — Supplementary Information. [file 41598_2022_6132_MOESM1_ESM.pdf]

SUPPLEMENTARY INFORMATION

Gadolinium-oxide nanoparticles for cryogenic magnetocaloric application

A. Zeleňáková[a]*, P. Hrubovčák[a,b], A. Berkutova[a], O. Šofranko[a], N. Kučerka[b,c], O. Ivankov[b,d], A. Kuklin[b,e], V. Girman[a], V. Zeleňák[f]

[a]*Institute of Physics, P.J. Šafárik University, Park Angelinum 9, 04001 Košice, Slovakia*

[b]*Frank Laboratory of Neutron Physics, Joint Institute for Nuclear Research, Joliot-Curie 6, Dubna, 141980 Russia*

[c]*Comenius University in Bratislava, Department of Physical Chemistry of Drugs, Mlynská dolina, 832 32 Bratislava, Slovakia*

[d]*Institute for Safety Problems of Nuclear Power Plants NAS of Ukraine, Kyiv Ukraine*

[e]*Moscow Institute of Physics and Technology, Institutsky per. 9, Dolgoprudny, Moscow Region, 141700, Russia*

[f]*Institute of Chemistry, Faculty of Science, P.J. Šafárik University, Moyzesova 11, 041 54 Košice, Slovakia*

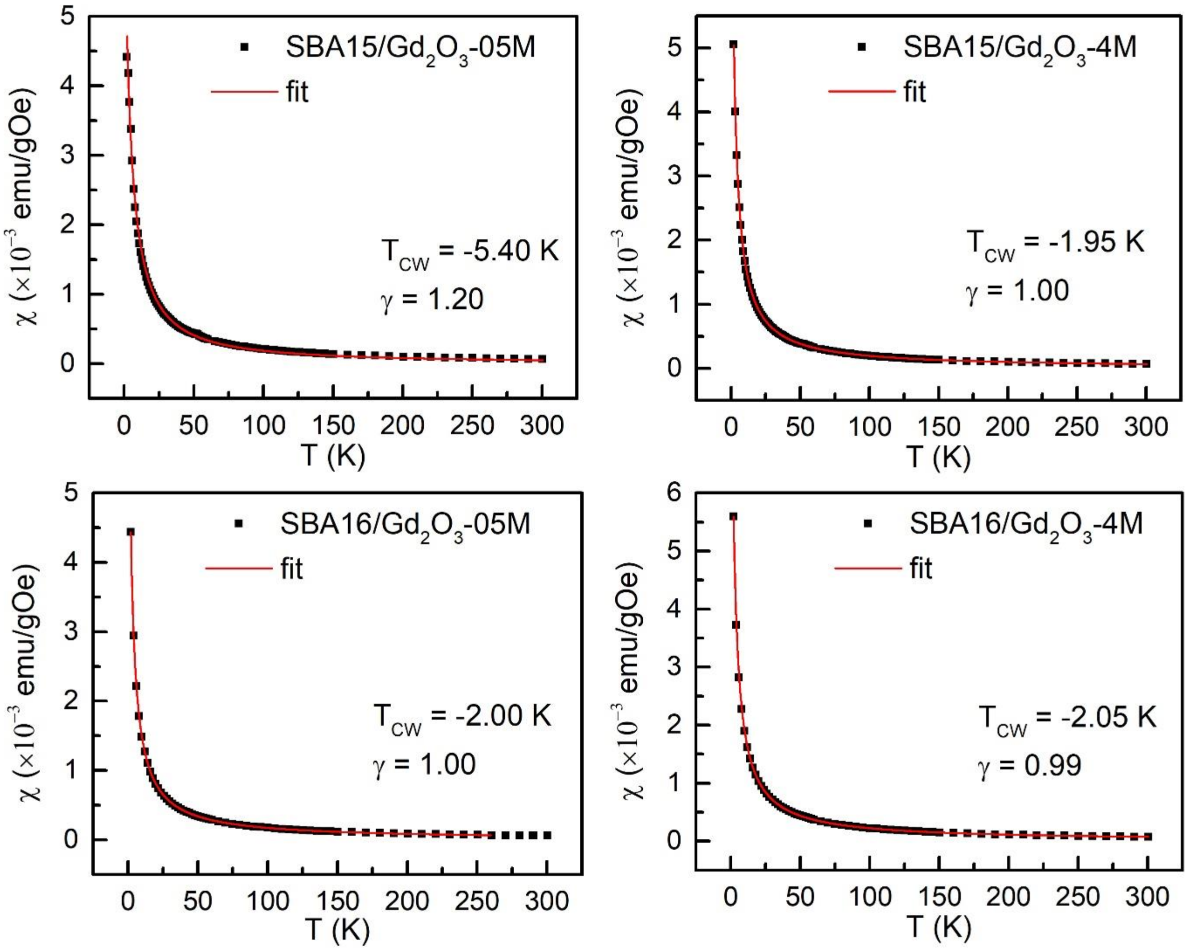

Supplementary Fig. 1 Susceptibility (black squares) of the systems SBA15/Gd_2O_3-0.5M; 4M and SBA16/Gd_2O_3-0.5M; 4M calculated from the magnetization obtained in ZFC regime in the applied static field 100 Oe. Red lines represent the best fits to the experimental data according to the Curie-Weiss law $\chi=C/(T-T_{CW})^{\gamma}$, where C and T_{CW} is Curie-Weiss constant and temperature, respectively.

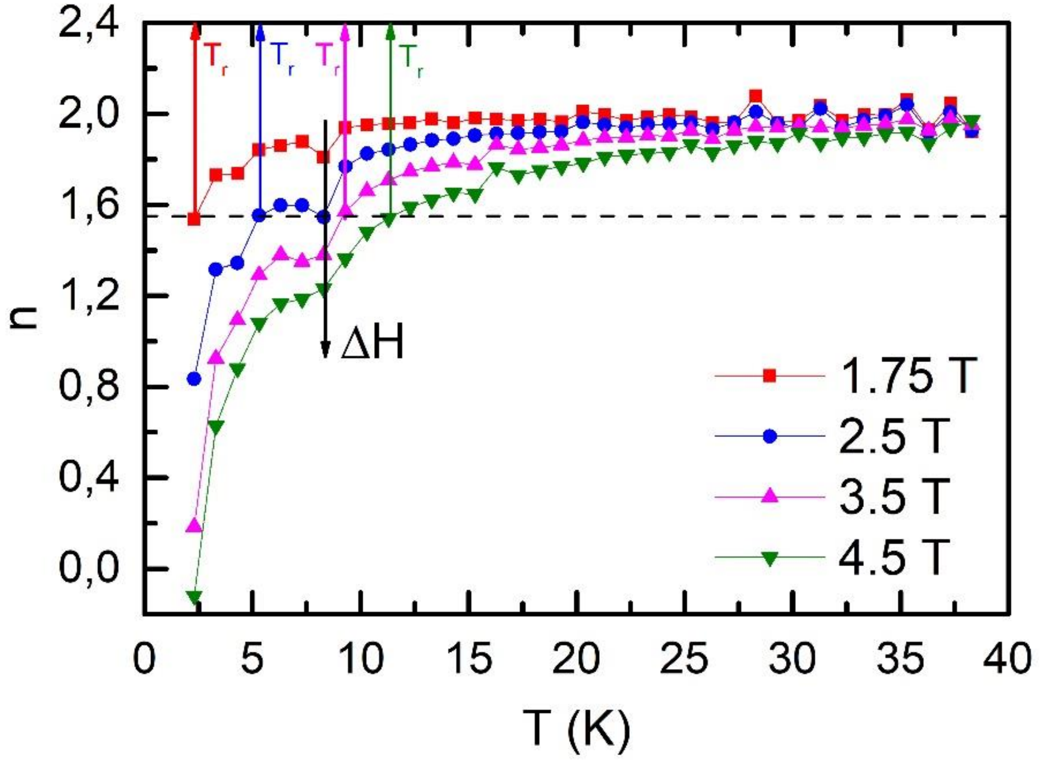

Supplementary Fig. 2 Temperature and applied field change dependence of the exponent *n* calculated for the system SBA16/Gd_2O_3-4M. The reference temperatures T_r have been determined as the temperatures corresponding to the value n = 1.55 (dashed line) of each *n(T)* curve. Black arrow indicates applied field change dependence of *n(T)* local minimum.
